# Microsphere Assisted Super-resolution Optical Imaging of Plasmonic Interaction between Gold Nanoparticles

**DOI:** 10.1038/s41598-017-14193-3

**Published:** 2017-10-23

**Authors:** Beibei Hou, Mengran Xie, Ruoyu He, Minbiao Ji, Sonja Trummer, Rainer H. Fink, Luning Zhang

**Affiliations:** 1 School of Chemical Science and Engineering, and Shanghai Key Laboratory of Chemical Assessment and Sustainability, Tongji University, Shanghai, 200092 China; 2 State Key Laboratory of Surface Physics and Department of Physics, Fudan University, Shanghai, 200433 China; 3 Physikalische Chemie II, ICMM, Friedrich-Alexander-Universität Erlangen-Nürnberg (FAU), Egerlandstraße 3, 91058 Erlangen, Germany; 4 CENEM, Friedrich-Alexander-Universität Erlangen-Nürnberg (FAU), Egerlandstraße 3, 91058 Erlangen, Germany

## Abstract

Conventional far-field microscopy cannot directly resolve the sub-diffraction spatial distribution of localized surface plasmons in metal nanostructures. Using BaTiO_3_ microspheres as far-field superlenses by collecting the near-field signal, we can map the origin of enhanced two-photon photoluminescence signal from the gap region of gold nanosphere dimers and gold nanorod dimers beyond the diffraction limit, on a conventional far-field microscope. As the angle θ between dimer’s structural axis and laser polarisation changes, photoluminescence intensity varies with a cos^4^θ function, which agrees quantitatively with numerical simulations. An optical resolution of about λ/7 (λ: two-photon luminescence central wavelength) is demonstrated at dimer’s gap region.

## Introduction

The resonant coupling of an incident electromagnetic wave with the energy levels of metallic nanostructures results in collective motion of conduction electrons in phase, which is called localized surface plasmon resonance (LSPR)^1–3^. The induced electron oscillation gives rise to strong absorption and scattering of light at resonant wavelengths. The energies of LSPR are highly sensitive to the micro-environment around the nanostructures, giving them intrinsic capabilities to report minute changes in their immediate surroundings, such as refractive index change^4–6^. Recent studies have shown that effects as small as single molecule binding on gold nanosphere (GNS) and gold nanorod (GNR) can be detected by monitoring the LSPR shifts^7,8^. When metal nanostructures are made to approach each other through applied force^9^, chemical bonding^10,11^, electrostatic interactions^12^, and aggregation, the interaction of LSPR results in change of electronic resonances^13^ and enhancement of local electric fields. The LSPR coupling in a metal nanostructure ensemble enables researchers to design and create artificial nanosystems with flexible optical properties. For example, ensembles of gold nanoparticles (GNP) in cells have been used to probe biological molecules through enhanced extinction spectra or Raman scattering^14^ Other spectroscopic techniques such as second harmonic generation and two-photon photoluminescence (TPPL) have also shown to exhibit enhancement from plasmonic interaction^15^, a feature highly desirable in molecular sensing. The interaction between nanoparticles as a signal enhancement mechanism has attracted much attention and is often used in the studies of adsorption and configuration of biological macromolecules at interfaces, such as surface enhanced Raman scattering (SERS)^16,17^, high-harmonic generation^18^, metal-enhanced fluorescence^19^, and two-photon photonluminescence (TPPL)^20,21^.

With vast potential for applications using plasmonic coupling, its full utilization calls for a better understanding of the correlation between the structure and its optical property. Therefore, techniques that can directly visualize the origin of optical signal with nanometer resolution are needed. Because of the near-field nature of surface plasmon within metal nanostructures, the evanescent wave that carries nanometer length scale electric field information cannot be picked up by far-field imaging methods. The noble metal nanoparticle dimer is among the most studied interaction models^22–24^. In recent years, several groups have sought to visualize plasmonic coupling with scanning near-field optical microscope (SNOM), which can overcome the optical diffraction limit^25–28^. Nanometer-resolved Raman and TPPL images from gold nanoparticle dimers and trimmers have been mapped by SNOM. Results have shown that signals originate from the gap region in certain structures. However, limited scan speed and reproducibility in tip-sample interaction are major bottlenecks in SNOM, limiting its wider application in studying plasmonic interactions. In conventional far-field microscopy, spatial features of the nanostructures become lost when localized evanescent waves decay exponentially in medium. Recently, it is shown that dielectric microspheres may act as far-field superlenses to break the diffraction limit. When a microsphere is positioned close to a nanometer object, the photons at near field can be picked up and transformed into propagating waves in the far field so that super-resolution imaging is achieved^29,30^. A resolution of λ/17 was reported for microsphere-assisted imaging on a laser scanning confocal microscopy^31^. However, the resolution in the study was calculated by the edge-to-edge separation of the bright spots from gold nanodots, which gave a much higher resolution than conventional point spread function (PSF) analysis. Since 2012, a series of studies have shown that when high refractive index microspheres are submerged in a liquid, a typical resolution of λ/7 is achieved^32–34^. The method was later called submerged microsphere optical nanoscopy (SMON), and has been successfully applied to various samples such as cells^35^, and viruses^36^.

In this Article, we demonstrate how submerged microsphere in tandem with nonlinear optical microscopy can directly map the origin of enhanced TPPL at the gap regions of gold nanospheres (GNS) and gold nanorods (GNR) dimers. The intrinsic TPPL signal from interacting gold nanoparticles allows us to elucidate the relationship between dimer structure and local plasmon response with super-resolution. Furthermore, we apply finite-difference time-domain (FDTD) methods to simulate the local field enhancement, aiming to gain insight of the plasmonic coupling within nanostructures. Our numerical simulations show quantitative agreement with optical measurements. This study illustrates that through a far-field microscope, it is possible to look into the nature of plasmonic interactions using the TPPL signal from unlabeled GNSs and GNRs in dimer configuration.

## Results

**Figure 1:**
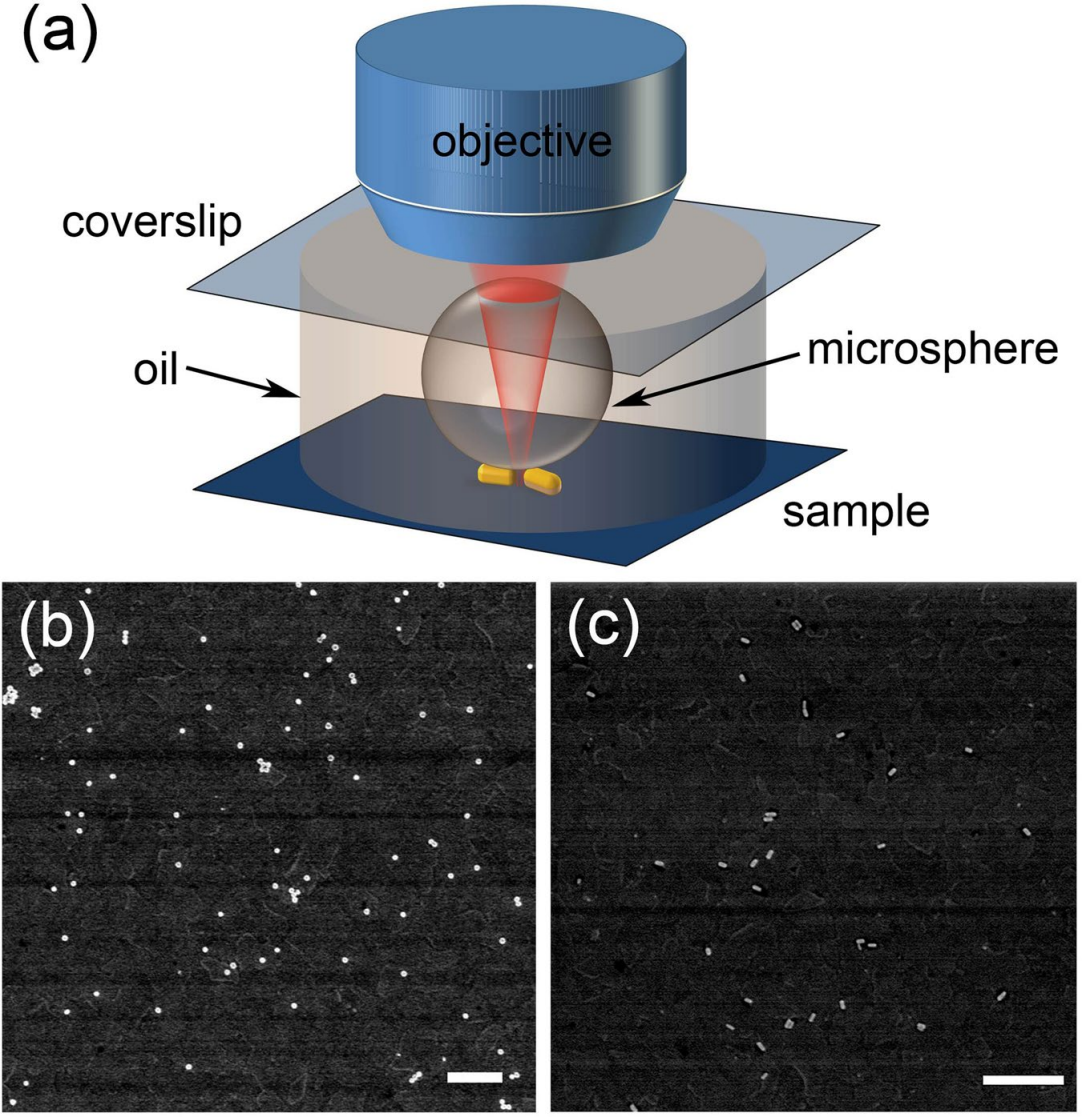
(**a**) Schematic of experimental setup. The microspheres are BaTiO_3_ with refractive index larger than 1.93. Drawing is not to scale. SEM images of gold nanospheres (**b**) and gold nanorods (**c**) samples. Scale bars: 1000 nm.

The schematic in Fig. 1(a) shows the experimental geometry for imaging. Ideally one would desire to have the same area imaged by both optical and scanning electron microscopy (SEM). However, deposition of BaTiO_3_ microspheres on top of the GNP sample is a random process, without control of the location of the microsphere. Statistically speaking, microspheres can appear at regions with some single and dimer structures under them. The SEM images of nanosphere and nanorod samples are shown in Fig. 1b and c. The majority of the GNPs are very well dispersed, appearing as single particles. A small fraction of the nanoparticles appear as dimers and multimers. These features in SEM are consistent with what we observe in TPPL imaging, as presented below.

### Imaging and analysis of gold nanospheres

**Figure 2:**
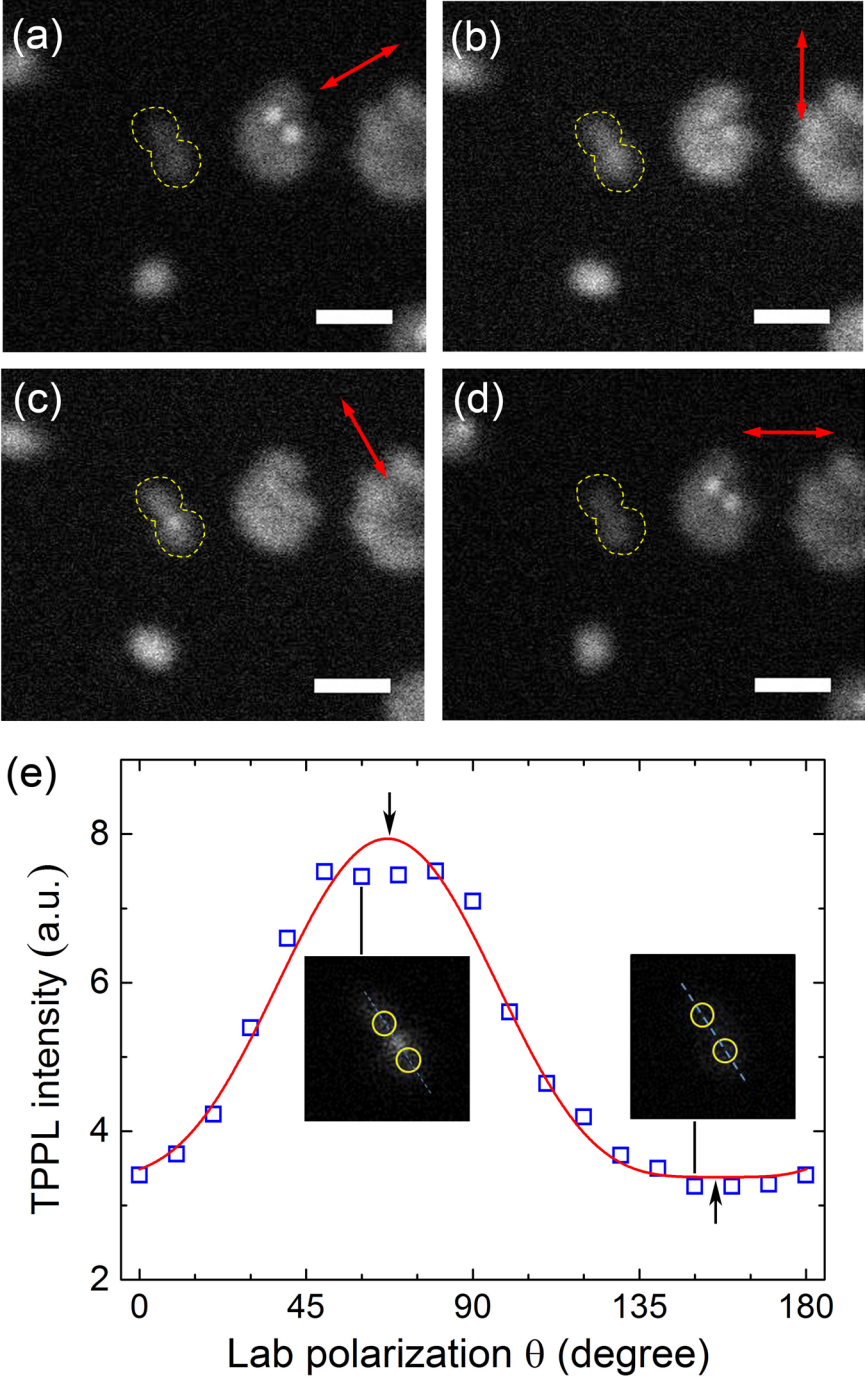
(**a**–**d**) TPPL images of the GNSs under BaTiO_3_ microsphere with different incident polarisation directions (indicated by red arrows). Yellow dotted lines indicate the region where signal from a dimer structure is integrated. All scale bars: 500 nm. (**e**) Integrated TPPL intensity versus incident polarisation (open squares) together with a cos^4^θ curve fitting (solid line). Insets are images recorded at two polarisations close to the fitted maximum and minimum (black arrows). Yellow circles in the insets indicate the estimated topography of the GNS dimer.

Gold nanospheres are readily visible in the TPPL images as shown in Figs 2a–d, S1, and S2. In order to quantify the sizes of important features, we use rigorous mathematical analysis to determine the point-spread function (PSF)^37–39^. Three types of TPPL features can be identified: (a) individual spots with PSF widths of 184 ± 26 nm, (b) clustered spots with sizes ranging from 400 nm to 1000 nm, and (c) spots with higher brightness within the clustered regions of (b) and PSF widths of 86 ± 18 nm. When we use SEM to image some samples, GNSs and GNRs show up as isolated single nanoparticles as well as clusters (dimer, trimer, etc.). This indicates that in the TPPL results individual spots of type (a) originate from single nanoparticles, and clustered features of type (b) come from oligomers. Unlike fluorescent molecules and quantum dots, most GNSs are able to give relatively strong photoluminescence without obvious bleaching and blinking. We take caution to maintain a low laser power on the samples to prevent potential photo-damage^40^.

A GNS dimer is present in Fig. 2, outlined with dotted line. In order to quantify the PSF widths of individual GNSs and dimer structure, we need to consider the magnification factors introduced by the microspheres (see SI, Figs S3, S4). The nanostructures on blu-ray disc are used as length scale standard, and the magnification factors of BaTiO_3_ microspheres with different diameters are deduced. A magnification factor of about 2 is observed and is comparable to formula derived from geometric optics reported before^37,41,42^. Although these results in SI have been reported previously, we repeat these processes so as to verify the feasibility of our imaging system^34,41^. In TPPL images, individual GNSs have PSF widths of about 184 nm (Fig. S5a), which are larger than the 100 nm ± 8 nm diameter measured by SEM. Two factors may render the image size larger than the actual nanoparticle size, which are: partial collection of the evanescent wave by microsphere, and exceedingly large scattering cross section of GNSs within the TPPL spectral region. First, dielectric microspheres may not collect all the near-field photons in a strict sense. Along with the evanescent wave from GNSs, contributions from propagating wave photons may also be collected. Similar phenomenon has been observed previously that near-field images from SNOM are actually larger than the actual nanoparticle size^27,28^. In addition, gold nanoparticles are known to exhibit strong scattering near their LSPR. The TPPL emission spectrum of our sample encompasses GNS’s LSPR that centers at 540 nm (diameter = 100 nm)^43^. Simulations have found that the scattering cross section of GNSs is about 1.5 times larger than that of a dielectric nanoparticle^44,45^.

Bright TPPL features of type (c) having PSF widths in the range of 70–100 nm appear within dimer and oligomer, as seen in Fig. 2a–d. They appear to be smaller than individual GNSs and come from the gap regions, where coupling occurs between adjacent GNSs. In order to quantify physical parameters of the dimer in Fig. 2a–d, PSF analysis is implemented. First, we obtain a gap distance of ~83 nm by PSF analysis of the peaks distance between the two nanoparticels (Fig. S5b). Assuming that the centroid of the PSF fitting corresponds to GNS center, the dimer’s edge-to-edge distance is calculated by subtracting GNS’s diameter from the center-to-center distance. The 83 nm gap distance should not be confused with the optical resolution. In the following results, we show that at the gap region of a GNS dimer when plasmonic coupling is strongly localized and the electric field is greatly enhanced, the TPPL signal at the gap can be imaged with a better resolution than that of individual GNS (184 nm, ~λ/3).

Upon PSF analysis, the TPPL signal at dimer’s gap region shows a FWHM of about 70 nm (Fig. S5b). On one hand, this is much smaller than the resolution obtained on individual GNS and it is likely due to the localized electric field enhancement at the gap. On the other hand, the 70 nm TPPL feature is not equivalent to the dimer’s physical gap distance. This is because TPPL mapping at the gap probes the local field enhancement, no the physical distance of the dimer. These features from the dimer not only appear narrower, but also exhibit intensity variation as the excitation laser polarisation changes. The signal is the strongest when the incident polarisation is parallel to interparticle axis, and the weakest when it is perpendicular. Integration of the TPPL intensity within the periphery of the dimer in Fig. 2a–d gives us the overall signal due to plasmonic coupling at the gap. A series of images with different laser polarisations (Fig. S2) is quantitatively analyzed. Plot of the TPPL intensity versus polarisation directions is shown in Fig. 2e. The horizontal axis “θ” is the angle between y-direction in the laboratory-frame and accumulated rotation angle of laser polarisation. Each point comes from integrated signal within the yellow periphery of the dimer. A fitting function with the form of I = a*cos^4^θ + b (I: signal intensity; a and b: constants) can fit the trend of the data points. Previously, researchers have confirmed that the TPPL intensity of GNP dimers has cos^4^θ dependence on the excitation polarisation^46–48^. Although we do not achieve actual sizing for individual GNSs (184 nm in image versus 100 nm actual diameter), a subwavelength resolution of ~λ/7 is achievable for imaging the gap regions of dimers (λ is about 540 nm as the center of TPPL emission spectrum). Numerical simulations are used to verify our experimental results.

**Figure 3:**
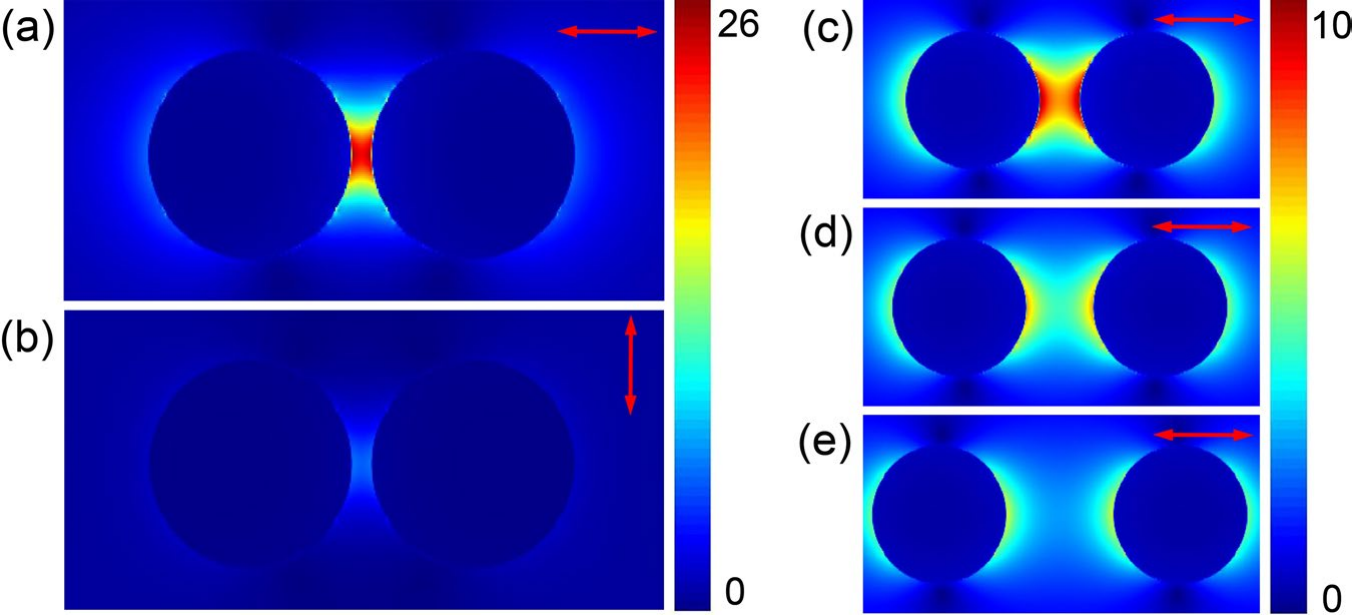
Plots of light field magnitude (|E|, λ = 800 nm) of a GNS dimer structure with incident polarisation (**a**) parallel and (**b**) perpendicular to the interparticle axis and a 10 nm gap, (**c**–**f**) parallel to the interparticle axis and a gap of (**c**) 30 nm, (**d**) 50 nm, and (**e**) 80 nm, respectively. Arrows indicate incident light polarisations.

We use the FDTD method to map the distribution of the electric field amplitude (denoted as E-field, |E|) around the GNS dimer excited by 800 nm light. For a gap of 10 nm, Fig. 3a and b give the local electric field amplitude for two different incident light polarisations. Figure 3a shows that when light is polarised parallel to the dimer’s interparticle axis, the electric field in the interstitial site becomes the strongest. On the other hand, the local field at the gap is the weakest when the incident field rotates by 90 degrees. The strength of local E-field for gap widths of 30 nm, 50 nm, 80 nm in the dimer is given in Fig. 3c–e. Here one finds a decrease of the electric field with the increase of the distance between two GNSs. Plasmonic coupling between the nanospheres becomes less prominent as the gap increases to 80 nm. However, local field amplitude still gets enhanced at this separation. Experimental results shown in Figs 2 and S2 indicate moderate signal enhancement at the dimer’s interstitial region. Since the edge-to-edge distance of the dimer is estimated to be ~83 nm, simulations suggest that TPPL can be enhanced at this distance. We conclude that the specific GNS dimer in Fig. 2a–d has a relatively large gap, probably in the range of 80–90 nm, as suggested by both experiments and simulations.

### Imaging and analysis of gold nanorods

**Figure 4:**
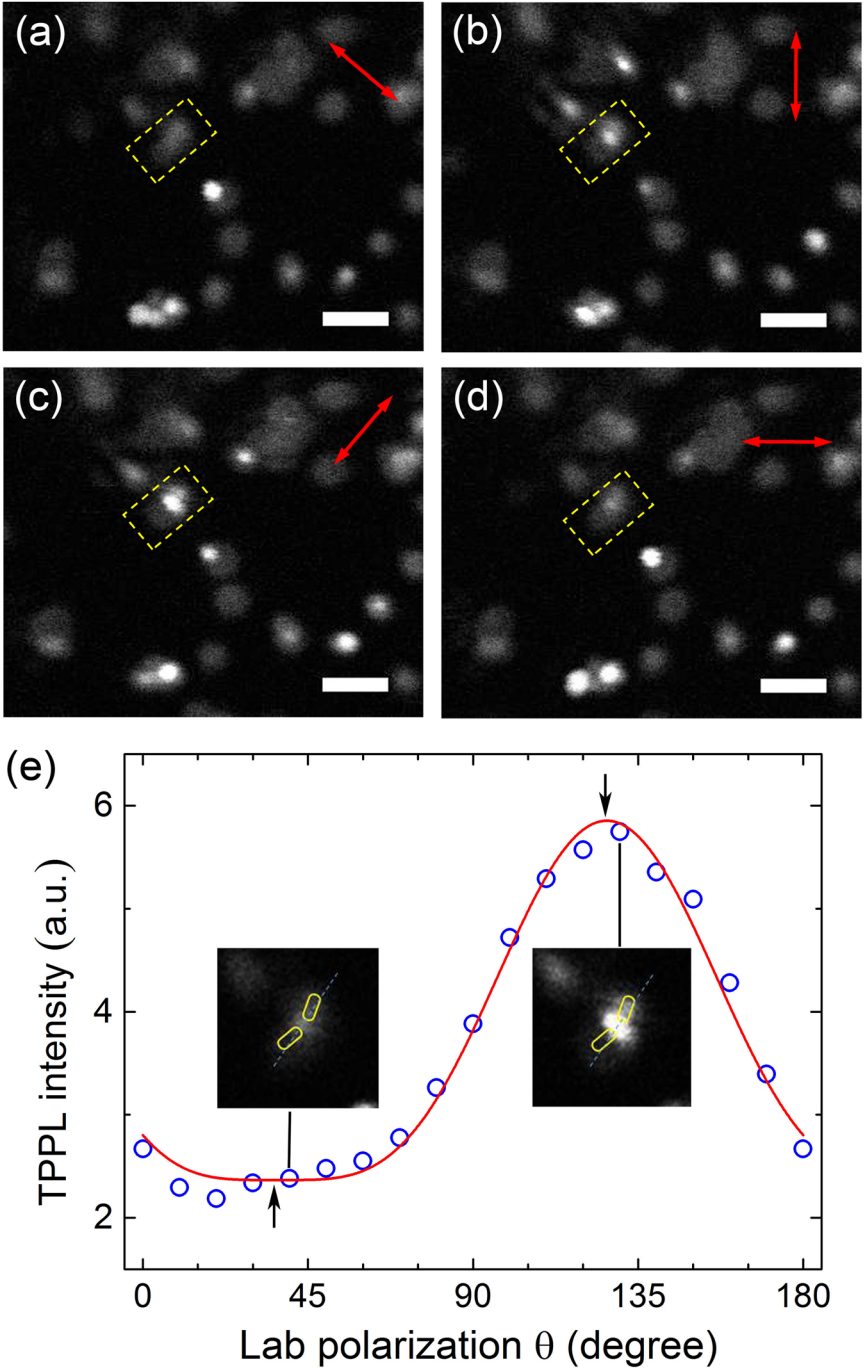
(**a**–**d**) TPPL images of GNRs imaged through BaTiO_3_ microsphere at four representative incident polarisation directions (indicated by red arrows). Yellow outline indicates the region where signal of a nanorod dimer is integrated to plot (**e**). All scale bars: 500 nm. (**e**) Integrated TPPL intensity at different incident polarisations (open dots) with a cos^4^θ curve fitting (solid line). Insets: Corresponding TPPL images close to the fitted maximum and minimum (black arrows), along with the estimated topography of GNR dimer.

GNR dimers have many possible configurations which differ from GNS dimers, in which the gap width is the only adjustable parameter. For GNR dimer, plasmonic coupling is affected by the distance and relative axial direction of nanorods, two parameters that we want to derive through experiments and numerical simulations. First, we study the polarisation dependence of TPPL so as to understand the local signal enhancement as a function of electric field direction. We then use the FDTD method to simulate representative configurations of GNR dimers aiming to elucidate the localized E-field distribution. Comparisons between simulations and the super-resolved TPPL images are made. Figures 4a–d and S6 show several representative images in which three types of distinctive features are seen: (a) isolated spots with PSF widths in the 200–270 nm range; (b) clusters that are twice or several times larger than the individual spot; and (c) very bright spots appear within regions of type (b). The extremely bright features of (c) are mostly circular shaped with some spots slightly elliptical, both with PSF widths in the range of 90 ± 10 nm. Similar to GNSs, individual spots (a) and clustered spots (b) come from single GNR and their clusters, respectively. Like GNSs, we cannot directly resolve the actual size of GNR (d = 40 nm, L = 96 nm) due to the fact that the TPPL signal collected by microsphere may have contributions from non-evanescent photons, rendering the signal not strictly from the physical near field. Large scattering cross section of GNRs may also contribute to larger measured spot size. In general, GNRs have stronger TPPL signal than GNSs, due to the anisotropic feature of nanorod that could increase the absorption cross section and the TPPL quantum yield of GNRs^49^.

The results of polarisation-dependence reveal some unique features of GNR which are different from GNS. First, analysis of Figs 4 and S6 shows that the TPPL intensity of a single GNR exhibits a cos^4^θ dependence on laser polarisation (θ is the angle between the polarisation direction and nanorod longitudinal axis, see Fig. S7). This is very different from single GNS, whose signal intensity does not vary with polarisation. The dependence on polarisation for single nanorod has been observed both in TPPL and photothermal studies^50^. It is also observed that the TPPL signal of a single GNR is uniform within its image periphery, without particularly bright regions. Clusters of GNRs are very different from single GNR. At certain laser polarisations, specific regions within the clusters become much brighter than the surroundings, which also results in large maximum/minimum signal ratio (Fig. S8). This is likely due to the enhanced plasmonic interactions between GNRs at specific electric field directions. In Fig. 4 (see Fig. S6 for details), an elongated cluster is observed with its central area intensity dependent on the incident polarisation. Based on the overall PSF width of the spot and its elongated shape, as well as the enhanced signal at the gap region, we assign this feature to a GNR dimer. The following analysis confirms this conclusion and helps to derive more information about the geometry of this dimer.

**Figure 5:**
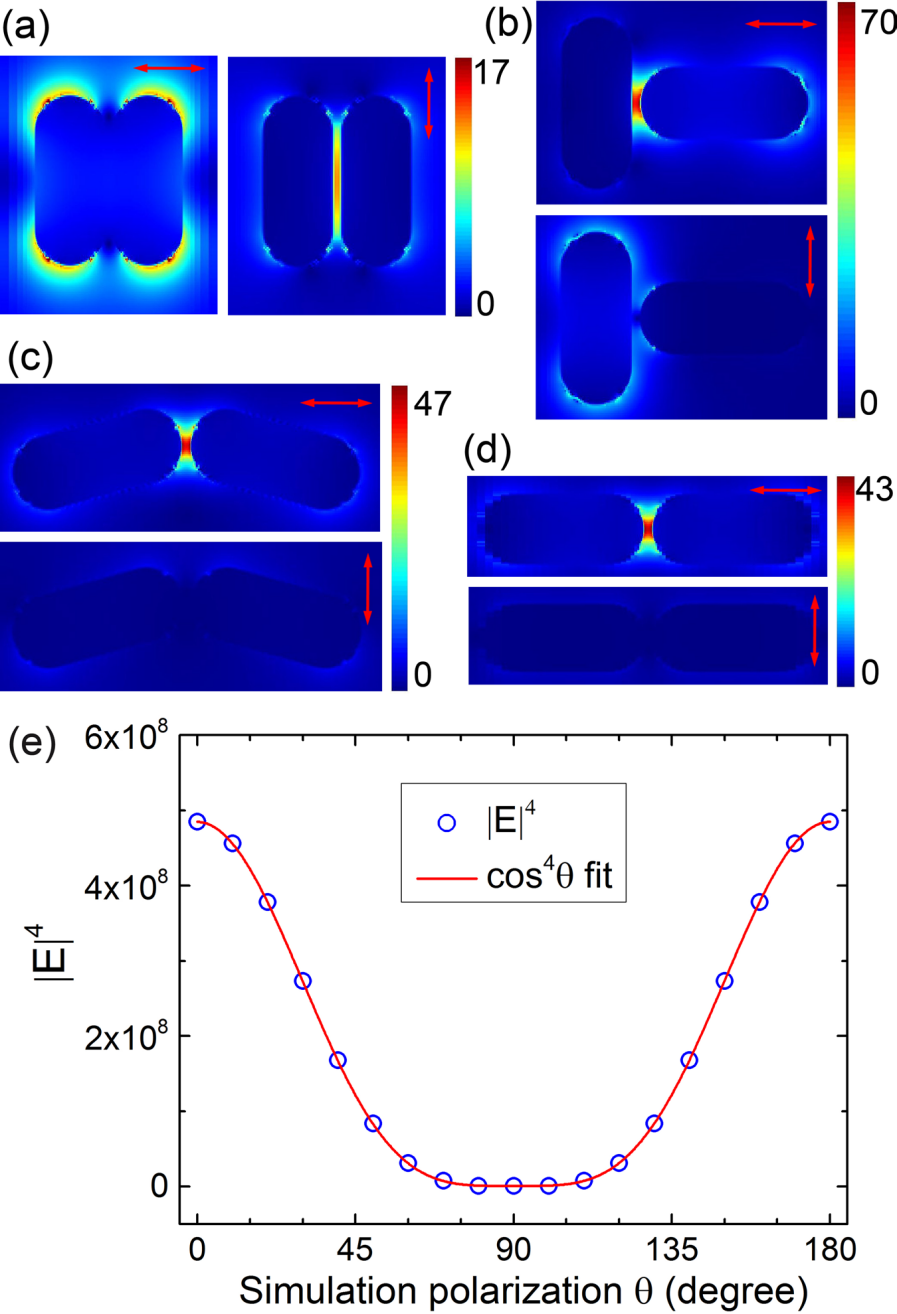
EM field intensity enhancement contours of different GNR dimers for (**a**) side-to-side configuration, (**b**) T-shaped configuration, end-to-end configuration with δ of (**c**) 150° and (**d**) 180° calculated using FDTD. The red arrows indicate the incident polarisations. (**e**) The value of |E|^4^ obtained for a dimer with δ = 150° (as in (**c**)) at different laser polarisations along with a cos^4^θ fitting curve.

Image analysis of the dimer (Figs 4 and S6) shows that its intensity depends on the laser polarisation in a cos^4^θ function, like individual GNRs discussed above. When the laser polarisation is nearly perpendicular to the supposed interparticle axis, the gap region signal becomes the weakest (Fig. 4a). As the laser polarisation changes, signal from the gap region starts to increase. The signal gradually increases to maximum value when the laser is polarised along the supposed interparticle axis (Fig. 4c). Its intensity variation can be fitted with a cos^4^θ function as shown in Fig. 4e. Simulations on various dimer geometries are then carried out to confirm the experimental data. First, we simulate the E-field of typical structures such as side-by-side and end-to-end, in which GNRs form parallel and linear structure, respectively. Perpendicular geometries such as the “L and T configurations” are also simulated. In the end-to-end structures, we also vary the relative angle between two GNRs’ main axes. The simulation results are shown in Figs 5 and S9. The local E-filed helps us understand where the TPPL signal should be enhanced experimentally, which can then be used to deduce the geometry of the dimer.

Using parameters similar to the experiments, simulations first rule out the “side-by-side” configuration in Fig. 5a. In this case, the E-field in between the two nanorods is the strongest when light is polarised perpendicular to the interparticle axis (dotted line). On the contrary, the experimental signal reaches maximum when light polarisation is along the interparticle axis (i.e., Fig. 4c). For the “T configuration” in Fig. 5b, E-field at the gap region is the strongest when light polarisation is parallel to the interparticle axis. However, the E-field becomes highly asymmetric when the light polarisation is switched to perpendicular. This would result in large spatial redistribution of the TPPL intensity within the dimer. Thus far, this geometry does not agree with experiments, in which we see no obvious intensity shift between GNRs with changing polarisation. Experimentally, TPPL intensity at the gap of the GNR dimer increases or decreases without redistribution of the surrounding signal intensity. Simulations on the “end-to-end” configurations are given in Fig. 5c,d and show agreement with experiments. For these geometries, the |E|^4^ of the interstitial region follows a cos^4^θ function with θ being the accumulated rotation angle between dimer’s interparticle axis and the input light polarisation, as shown in Fig. 5e. These simulations agree very well with the quantitative analysis of Figs 4 and S6.

In the end-to-end geometries, we may define the angle between the longitudinal axes of two nanorods to be δ. As we simulate different geometries with δ ranging from 60° to 180°, the central gap region always has enhanced |E|^4^ which also follows a cos^4^θ function. With increasing δ, the enhancement factor at dimer’s gap region becomes larger. Currently, we cannot determine the precise angle δ between the two nanorods because only a qualitative relation between experiment and simulation has been established. In the simulation in Fig. 5e, |E|^4^ may reach zero. However, the experimental TPPL signal of a dimer does not diminish at all polarisations. The minimum TPPL signal level is also affected by the noise level of the instrument and the lower limit of gray scale images. Nevertheless, end-to-end dimer configuration can be inferred from the experimental results beyond reasonable doubt.

## Discussion

Apparently, resolution is the vital determinant of the performance of an imaging system. In this paper, the resolution of ~70 nm is obtained by PSF analysis of the region of enhanced electric filed between the GNSs in a dimer. Interestingly, the resolution is at the best ~184 nm when analyzing the PSF of individual GNS with a nominal diameter of 100 nm. Similar results are also seen in the case of gold nanorods. The two different resolutions seem inconsistent at first, but one may rationalize and interpret as the following. In the case of individual nanoparticles, TPPL spots are larger than the physical dimensions of the GNS or GNR by about 1.8 times. This is likely caused by the partial collection of the evanescent wave by the microspheres and relatively large scattering cross section at the TPPL wavelength. In the case of imaging of the dimer’s gap region, it is reasonable to assume that the region with electromagnetic field enhancement is comparatively small than the physical size of the gap and the particle diameter. The region with strongest TPPL enhancement is highly local and may be approximated as a point-dipole. In this case, we reach a resolution of λ/7, which is most likely the intrinsic resolving power of our system. In a study on second harmonic field enhancement on nickel nanobricks, electric field was also found to become localized in specific regions smaller than 100 nm^51^. This further supports our argument.

We want to point out that the results in Fig. 5 are not all the coupling modes within the GNR dimer. In linear optical spectroscopy, recent simulations show that different plasmonic coupling modes can affect the optical image obtained through microspheres^52^. Coupled plasmonic modes are also important in TPPL systems, so we perform further simulations at different wavelengths (details in SI and Fig. S9). We find that plasmonic coupling is enhanced at two spectral ranges on a GNR dimer, which are at 625 nm and 800 nm, the latter one being our experimental wavelength. The two spectral features are similar to previously reported spectra of GNR dimers^53^. Simulations show that a hot spot is generated in the gap region with excitation at both wavelengths. Both cases exhibit polarisation dependence as the experimental data. However, excitation at 800 nm generates stronger amplitude of the E-field than at 625 nm. Experimentally, this may contribute to better image contrast at 800 nm. Although we have not carried out experiments with 625 nm excitation, we believe that our 800 nm laser is quite appropriate for the super-resolution imaging and contrast forming, although this wavelength is not the only choice. Future experiments at various wavelengths are expected to give deeper understanding of such plasmon system.

In addition, we are aware that photoluminescence imaging^54,55^ and photothermal imaging^50^ have also revealed polarisation dependence signal from nanowire/nanorod on far-field microscopes. We use the combination of far-filed microscopy with microsphere for the purpose to achieve super-resolution at nanosphere and nanorod dimer’s gap region. In our case, the polarisation phenomena help us find out the location and the size of the signal enhancement region in the dimers. Without submerged microspheres, we are not able to reach super-resolution at these locations.

Utilizing the near-field photon collection of BaTiO_3_ microspheres, we are able to show that a conventional far-field microscope can map the TPPL of individual gold nanospheres, nanorods, and dimer structures, with a spatial resolution of 70 nm at the dimer’s gap region. Without using fluorescent label, we can directly rely on the intrinsic two-photon photoluminescence of GNPs for imaging, and the super-resolved spatial distribution of signal reflects the strength and location of plasmonic coupling between nanoparticles. Using polarisation dependence studies and image analysis, we demonstrate that the TPPL intensity of a GNP dimer depends on the angle (θ) between the interparticle axis of GNPs and incident polarisation directions in a cos^4^θ function. These observations are also confirmed by FDTD simulations. In short, our method provides sub-diffraction visualization of the local field enhancement in coupled gold nanoparticles. Experimental observation of localized EM field enhancement in metal nanoparticle assemblies is not only important for fundamental research, but also may develop into new sensing technique in nanoscopy. The use of high refractive index micrsospheres in these fields may help to fulfill this task through sub-diffraction limited light delivery and collection at length scales important to nanotechnology.

## Methods

### Sampl preparation

Sample preparation begins with incubation of GNSs (diameter = 100 ± 8 nm, Nanoseedz) or GNRs (diameter = 40 ± 3 nm, length = 96 ± 7 nm, Nanoseedz) colloidal solution on coverslips pretreated with (3-Aminopropyl)trimethoxysilane. Electrostatic interactions between the silane functional groups and the colloidal particles result in the immobilization of nanoparticles on the surface. Afterwards, BaTiO_3_ microspheres (Cospheric) are put on the surface followed by addition of objective immersion oil (refractive index = 1.518) to create a liquid environment for the microspheres. The ability to achieve super-resolution for BaTiO_3_ microspheres (refractive index ~2.0) depends on the relative refractive index between the microsphere and its surrounding media. The final sample ready for imaging is a sandwich structure, consisting of the GNPs decorated coverslip at the bottom, with microspheres and immersion oil in between, and another coverslip on top.

### Instruments

For TPPL imaging, we use an upright laser scanning confocal microscope (Olympus BX61 with FV1200 scanning module), together with a femtosecond laser (Newport Insight Deepsee+, ~120fs pulse) centered at 800 nm for excitation. The laser passes through a half-wave plate in its beam path to allow polarisation control, then is focused using a water immersion objective (Olympus, UPLSAPO 60XWIR, NA = 1.2) through the top coverslip and microsphere onto the GNPs. The TPPL signal in the 475–750 nm range is collected by the same objective and further filtered by a 475 nm dichroic mirror and a 750 nm short-pass filter (refer to SI for more detail).

### Finite-difference time-domain (FDTD) simulations

The electric field distribution around GNP dimers is analysed by the FDTD method with a commercial software (FDTD solutions, 2016a trial version). The │E│ distributions are obtained by the following parameters. The gap distance between two 100 nm diameter GNSs is set as 10 nm, 30 nm, 50 nm and 80 nm, respectively. The GNRs have a separation of 5 nm which has a diameter, length and aspect ratio of 40 nm, 96 nm and 2.4, respectively, with a longitudinal plasmon resonance wavelength of 700 nm. The dielectric constant of gold is from John and Christy^56^. GNP dimers are placed 2 nm above a SiO_2_ surface and in an environment with refractive index of 1.518. A plane wave polarised laser of wavelength 800 nm illuminates perpendicularly onto the surface. In order to improve the simulation resolution with reasonable simulation time, the override mesh is set to be 1 nm for the entire region and 0.4 nm for the gap region.

## Electronic supplementary material

Supporting Information
