# Supplementary material for: Microsphere Assisted Super-resolution Optical Imaging of Plasmonic Interaction between Gold Nanoparticles
Source: Sci Rep. 2017 Oct 23;7:13789. doi: 10.1038/s41598-017-14193-3 (PMC5653755; doi:10.1038/s41598-017-14193-3)
Supplement: Supplementary file 1 — Supporting Information [file 41598_2017_14193_MOESM1_ESM.docx]

**Supplementary Information**

Microsphere Assisted Super-resolution Optical Imaging of Plasmonic Interaction between Gold Nanoparticles

Beibei Hou^1^, Mengran Xie^1^, Ruoyu He^2^, Minbiao Ji^2^, Sonja Trummer^3,4^, Rainer H. Fink^3,4^& Luning Zhang^1,*^

^1^ School of Chemical Science and Engineering and Shanghai Key Laboratory of Chemical Assessment and Sustainability, Tongji University, Shanghai 200092, China

^2^ State Key Laboratory of Surface Physics and Department of Physics, Fudan University, Shanghai 200433, China

^3^ Physikalische Chemie II, ICMM, Friedrich-Alexander-Universität Erlangen-Nürnberg (FAU), Egerlandstraße 3, 91058 Erlangen, Germany

^4^ CENEM, Friedrich-Alexander-Universität Erlangen-Nürnberg (FAU), Egerlandstraße 3, 91058 Erlangen, Germany.

Correspondence and requests for materials should be addressed to L.Z. (email: luningzhang@tongji.edu.cn)

**S1. Information of experimental procedures**

TPPL imaging in this paper is performed on a home-built TPPL microscope consisted of an upright microscope (Olympus BX61), a laser scanning confocal module (Olympus FV1200), and a tunable Ti-Sapphire ultrafast laser (Insight Deepsee+, Newport, CA). The laser has 120 fs pulse width and 80MHz repetition rate, with a wavelength of 800nm for this study. A quartz half-wave plate is placed in the excitation beampath to alter the polarisation direction of the incident light. Excitation laser is focused on the sample through a 60X water immersion objective (Olympus OPLSAPO, numerical aperture=1.2), and the photoluminescence signal is collected by the same objective. Signal is focused on a GaAsP detector (Olympus FV12-HSD) after passing through a 475nm dichroic mirror and a 750nm short-pass filter (detection range: 475nm-750nm). The power of the 800nm laser for GNSs and GNRs excitation is set at 1.5milliWatts and 3.0milliWatts, respectively. The white light images in the paper are acquired on an upright microscope (Olympus BH-2), equipped with a cooled CCD camera at -20 Celsius, and a halogen-tungsten lamp for illumination.

Image analysis is performed with ImageJ software. In all the data reported in the main text and the SI section, we use original acquired images for analysis without further adjustment of brightness and contrast. In several cases, only two types of image manipulations are used when needed, which are binning and smoothing. For example, in a few PSF analysis results given below, we use the smoothing function built within the ImageJ software. The smoothing process replaces each pixel with the average value of its 3×3 neighboring pixels.

**S2. TPPL images of all polarisations: GNSs sample in the main text**

The details of the two samples studied in this paper are given here. Fig. S1 is one of a TPPL image of GNSs, and an interception of the original image of the target area is shown in the picture. Fig. S2 shows the TPPL images of GNSs in 18 different polarisation directions. There is no processing for the images except the clipping procedure.


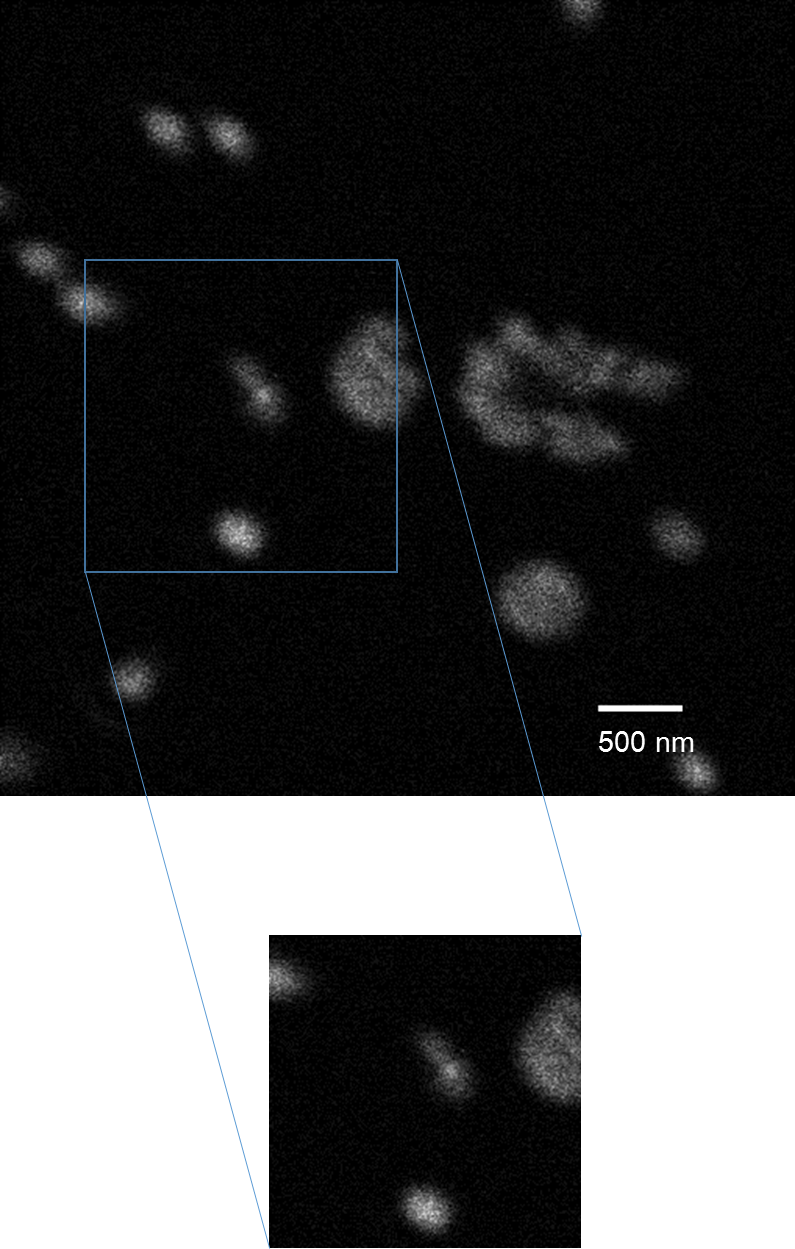


**Figure S1.** Representative TPPL image for gold nanosphere sample. The area with a dimer structure is enlarged. The sample is the same as Fig. 2 in the main text.


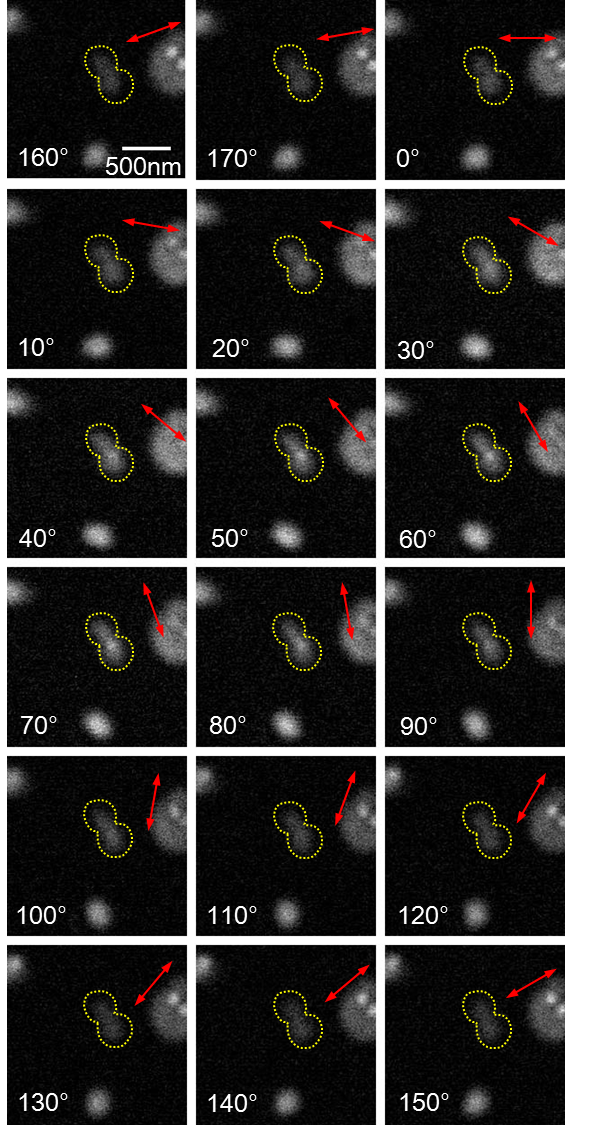


**Figure S2.** TPPL images of a GNS sample taken at different laser polarisation directions. Red arrows indicate laser polarisation. Yellow peripheries indicate the region within which dimer signal is integrated for analysis. Intensity of the dimer is shown in the main text in Figure 2e.

**S3. Blu-ray disc: SEM and white light image**

A scheme of microsphere nanoscopy is shown in Fig. S3(a). Figures S3(b) and (c) are SEM image and super-resolved white-light image of Blu-ray disc obtained through BaTiO_3_ (BTG) microsphere. The magnified regular lines on Blu-ray disc are clearly observed with the microsphere while these lines cannot be resolved without the microsphere.


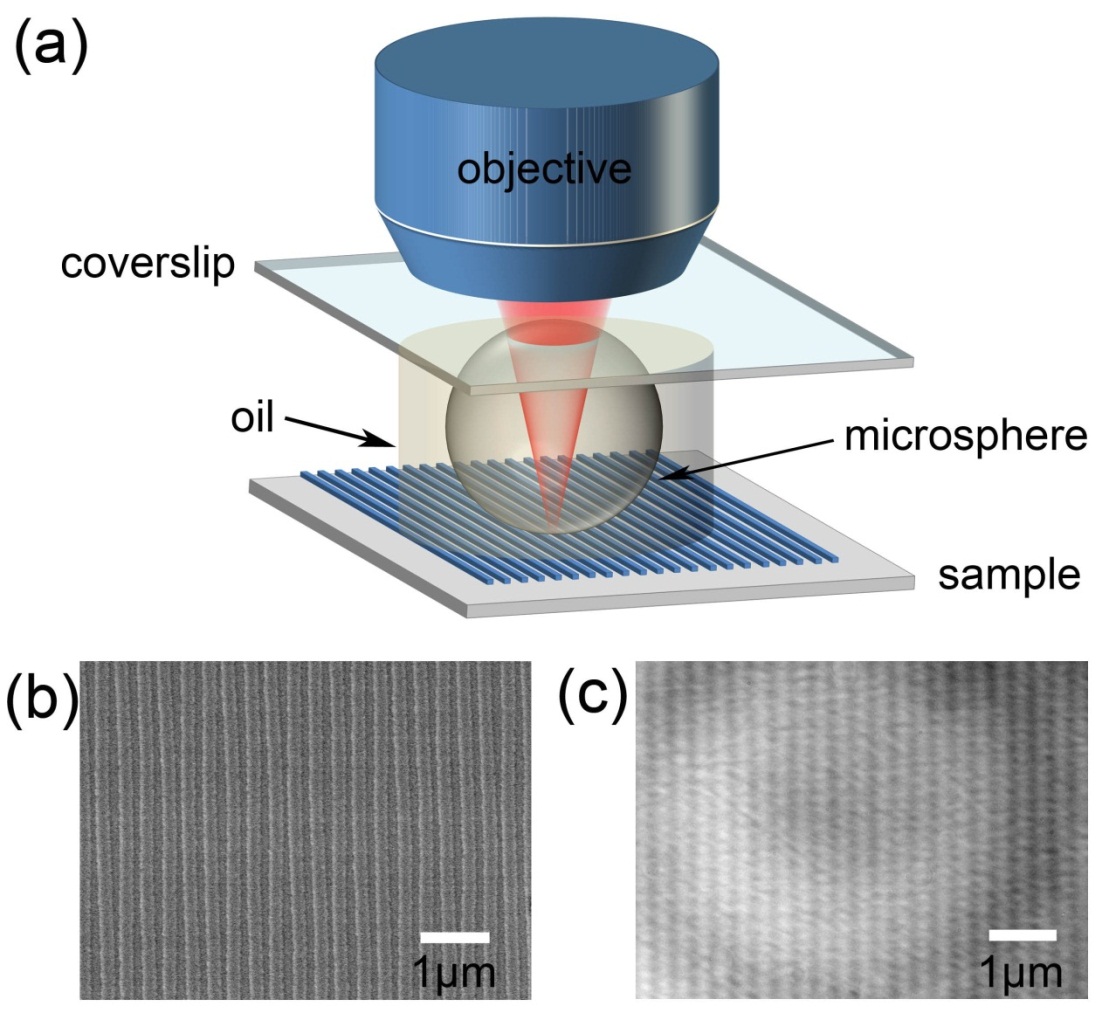


**Figure S3.** (a) Schematic of experimental setup. Drawing is not to scale. Images of the periodic lines on Blu-ray disc by (b) SEM and (c) SMON with a 100μm diameter BTG microsphere.

**S4. Magnification factor of BTG microspheres**

To calculate the magnification introduced by the BTG microspheres, we compare the feature size obtained on a length scale reference (10μm width lines) and those of Blu-ray disc. Since magnification factor of BTG microspheres is affected by the relative refractive index (n′) between BTG (n_sp_) and the immersion liquid (n_0_), all samples are investigated in the same immersion oil media as used in the paper, on 53 different BTG microspheres. The magnification factor(M) mostly falls within 1.9 to 2.6. The data can be roughly fitted with a linear function, as shown in Fig. S4. The overall trend is that the magnification is inversely proportional to the diameter of the BTG microspheres. The micron-scale microspheres used in this study can be approximated using geometrical optics. As for the theoretical formula, ray optics gives |M|~|n′/(2-n′)| for a spherical lens fully immersed in a media[^1-3^](#_ENREF_1), in which n′=n_sp_/n_0_=2.0/1.518=1.32 in our study. Thus, M is calculated to be 1.94 for our system. It can be seen that the measured M roughly agrees with the theoretical result from geometrical optics.


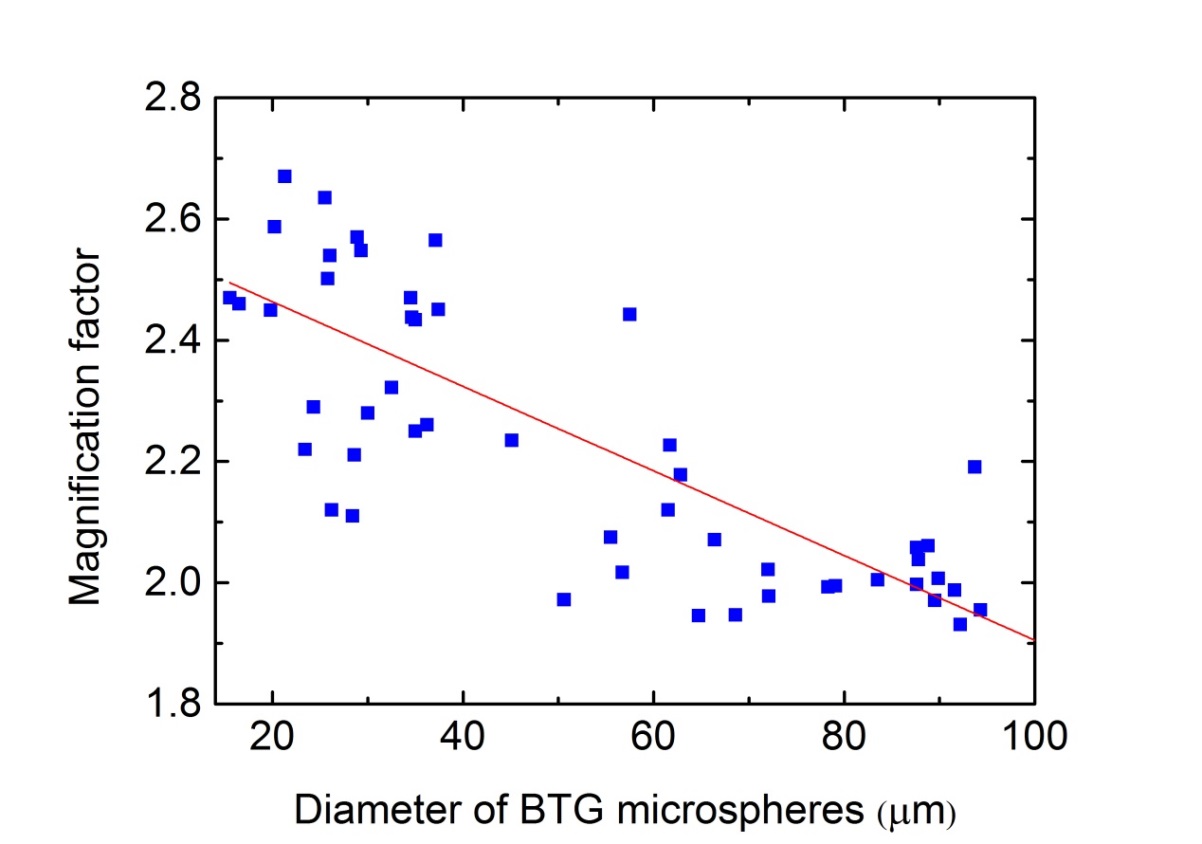


**Figure S4.** Magnification factors of BTG microspheres with different sizes.

**S5. Calculation of the gap between GNS dimer and the resolution of the system**

The PSF analysis for features of the size of individual gold nanoparticles is done by the following. In Fig. S5 (a), the signal intensity distribution of single gold nanosphere is fitted with a Gaussian function. This is repeated for a number of single GNS images. The FWHM of the fitting function is regarded as the TPPL imaging size, which is the PSF width for single GNS.

The PSF analysis for the dimer is done by the following. Prior to fitting, the image is smoothed to reduce background noise. The smoothing process replaces each pixel with the average of its 3 ×3 neighborhood. The image cross-section along dimer’s interparticle axis is fitted by Gaussian functions. In Fig. S5 (b), the black line is the experimental intensity profile measured along the red dash line in the inset. The black profile is fitted by three superimposed Gaussian peaks. We regard the centroid location of the left and right peaks as the center of two GNSs, so an edge-to-edge distance of 83 nm between the GNS dimer is calculated by subtracting diameter of 100nm from the distance between the two peaks. The FWHM of the middle peak is 70nm and should be not confused as the gap distance. The center peak originates from the electromagnetic field enhanced due to plasmonic coupling between the GNSs. Although the exact dimension of this enhanced region is not known, we may assume that the physical dimension is actually much smaller than the gap distance. The enhanced area which gives rise to the TPPL center peak can be approximated as a point dipole, and its size imaged by our method can then be regarded as the resolution.

**
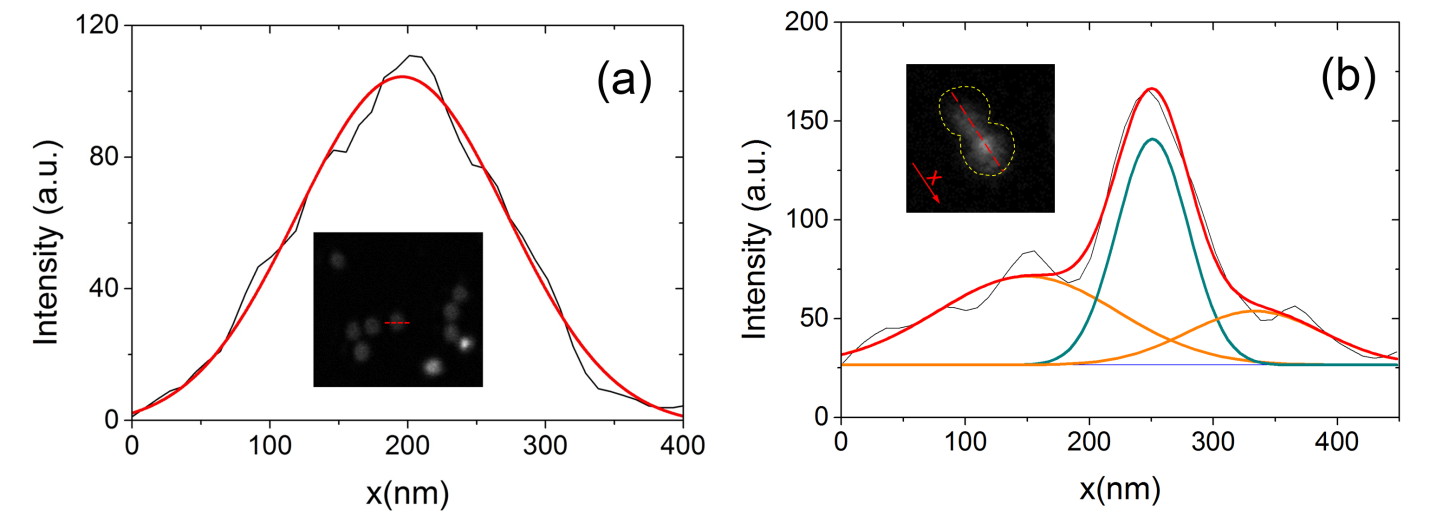
**

**Figure S5.** (a) The cross-section of an individual gold nanosphere (black line) and PSF analysis using Gaussian function fitting (red line). (b) The cross-section of gold nanosphere dimer and Gaussian function fitting of signal intensity. Insets are the corresponding TPPL images.

**S6. TPPL images at all polarisations: GNRs sample in the main text**

In the following, TPPL images of GNRs at 18 polarisation directions are shown in Fig. S6. There is no image processing except clipping of the originals.


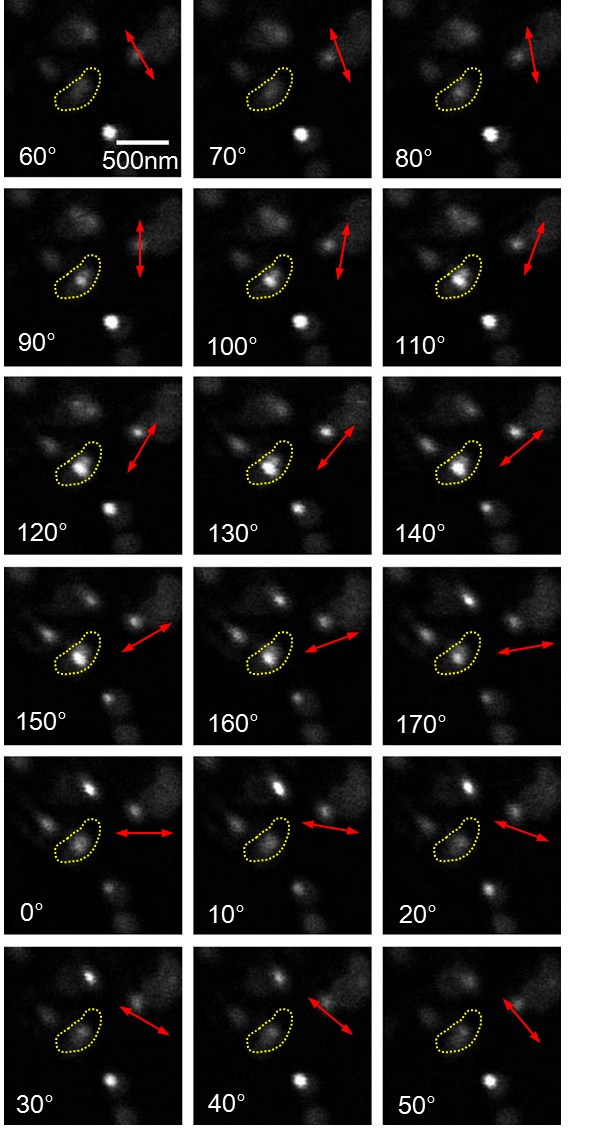


**Figure S6.** TPPL images of a GNR sample taken at 18 different laser polarisation directions.

**S7. Analysis of a single gold nanorod**

In Fig. S7 we show the TPPL intensity of a single GNR, which exhibits a cos^4^θ dependence on laser polarisation. Although the fitting function is similar to that from the gap regions, the ratio of the maximum signal to minimum signal is lower than that of the gap region. As the laser polarisation rotates, we analyze the maximum/minimum signal ratio of two types of TPPL spots, those from individual GNRs and those appear to be from the gap regions. This summary is plotted in Fig. S8. The lateral axis represents the size of individual bright spots (red solid circle) and spots within clustered regions (black solid square). The vertical axis represents the ratio of maximum to minimum integrated intensity of the spots. Spots within clustered regions have smaller sizes and a higher ratio than the individual spots. This helps us to distinguish two different types of TPPL features.

**
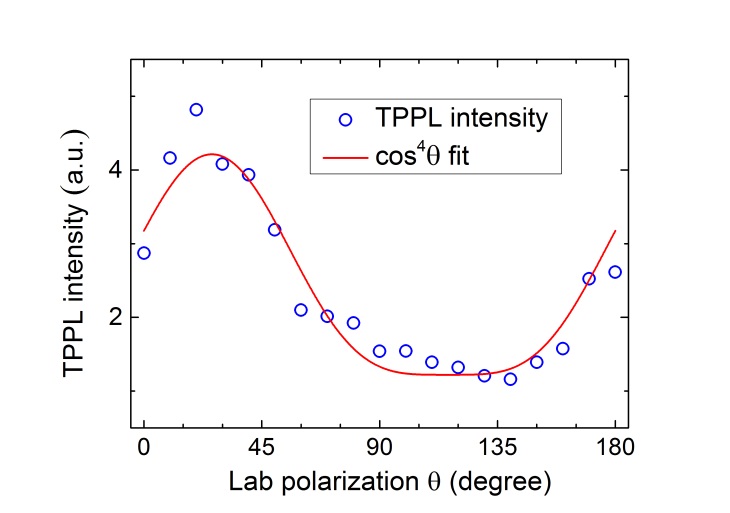
**

**Figure S7.** Integrated intensity of a single gold nanorod at different incident polarisations along with a cos^4^θ fitting curve.

**
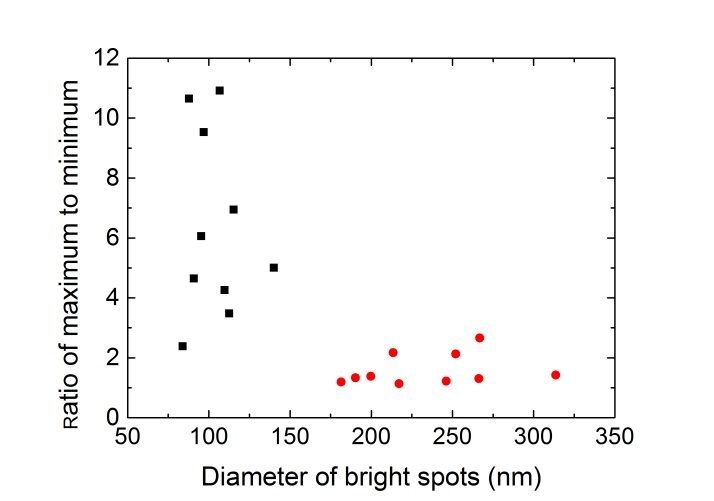
**

**Figure S8.** Plot of diameter of TPPL features along with their maximum to minimum TPPL signal ratio. Two types of features are shown.

**S8. Simulation of GNR dimer with different wavelength**

For the gold nanorod dimer with an end-to-end configuration, interactions can occur between the longitudinal and transverse modes of the rods. To study the dependence of electric field distribution on wavelength, we now show the simulation results with incident wavelengths of 800nm (strongest resonance band), 625nm (2^nd^ strongest resonance band), 700nm (LSPW of single GNR) and 525nm (TSPW of single GNR) in Fig. S9 (a-d). The results show that a “hot spot” is generated in the gap region at 800nm and 625nm, both of which show polarisation dependence. Comparison of Fig. S9(a) and (b) shows that excitation at 800nm generates a more intense electromagnetic filed, which may benefit image contrast in the experiment. No “hot spot” is observed at various polarisations at 525nm incident wavelength. Upon 700nm excitation, the electric field has a petal-like distribution around the nanorods and polarisation direction has no obvious influence on the field amplitude.

**
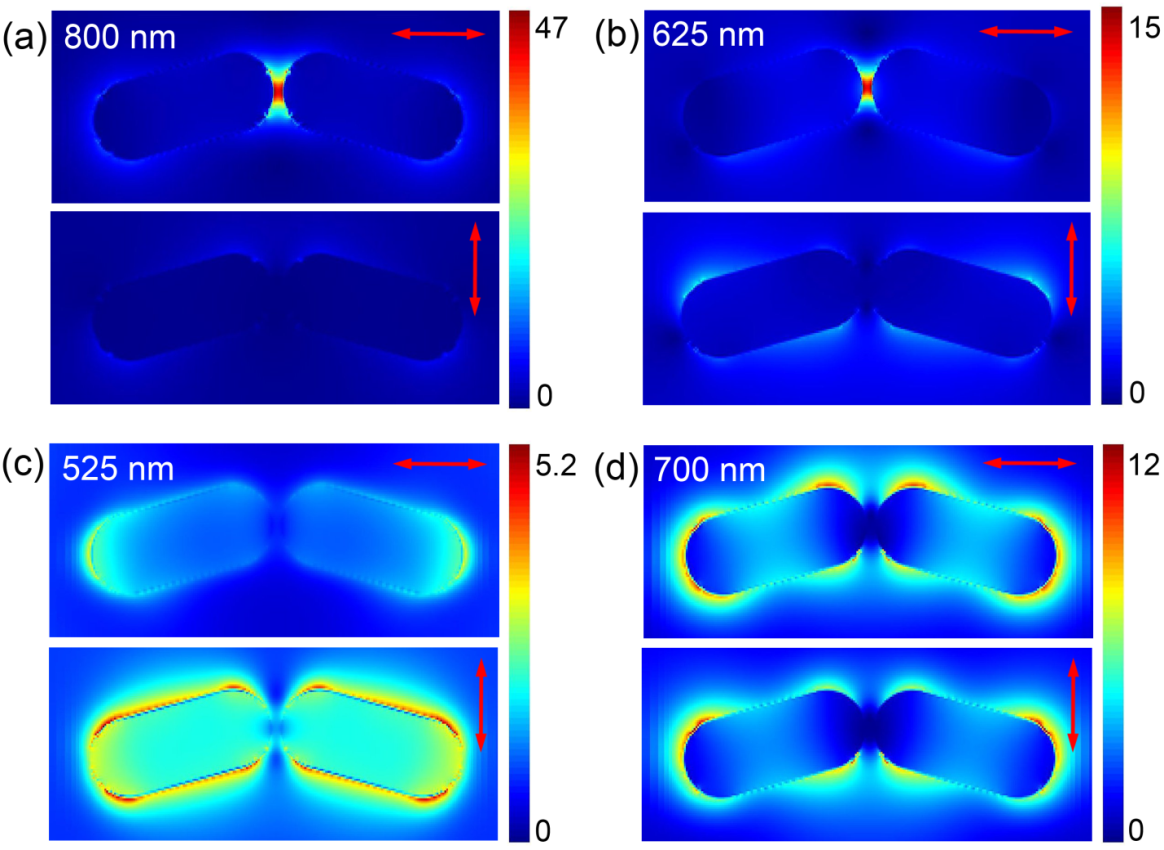
**

**Figure S9.** (a-d) Electric field distribution obtained from the FDTD calculations for the GNR dimer at excitation wavelength of 800nm, 625nm, 525nm and 700nm, respectively.

**References**

1. Darafsheh, A., Guardiola, C., Palovcak, A., Finlay, J. C. & Carabe, A. Optical super-resolution imaging by high-index microspheres embedded in elastomers. *Opt Lett* **40**, 5-8 (2015).

2. Allen, K. W. *et al.* Super-resolution microscopy by movable thin-films with embedded microspheres: Resolution analysis. *Annalen der Physik* **527**, 513-522 (2015).

3. Allen, K. W., Li, Y. & Astratov, V. N. Reply to “Comment on ‘Super-resolution microscopy by movable thin-films with embedded microspheres: Resolution analysis’ [Ann. Phys. (Berlin) 527, 513 (2015)]”. *Annalen der Physik* **528**, 901-904 (2016).
